# Physicochemical mechanisms of bacterial response in the photodynamic potentiation of antibiotic effects

**DOI:** 10.1038/s41598-022-25546-y

**Published:** 2022-12-07

**Authors:** Jennifer M. Soares, Francisco E. G. Guimarães, Vladislav V. Yakovlev, Vanderlei S. Bagnato, Kate C. Blanco

**Affiliations:** 1 São Carlos Institute of Physics, University of São Paulo, Av. Trabalhador São-Carlense, 400, 13566-590 São Carlos, São Paulo, Brazil; 2 Biomedical Engineering, Texas A&M University, College Station, TX USA

**Keywords:** Microbiology, Antimicrobials, Bacteria, Photobiology

## Abstract

Antibiotic failures in treatments of bacterial infections from resistant strains have been a global health concern, mainly due to the proportions they can reach in the coming years. Making microorganisms susceptible to existing antibiotics is an alternative to solve this problem. This study applies a physicochemical method to the standard treatment for modulating the synergistic response towards circumventing the mechanisms of bacterial resistance. Photodynamic inactivation protocols (curcumina 10 µM, 10 J/cm^2^) and their cellular behavior in the presence of amoxicillin, erythromycin, and gentamicin antibiotics were analyzed from the dynamics of bacterial interaction of a molecule that produces only toxic effects after the absorption of a specific wavelength of light. In addition to bacterial viability, the interaction of curcumin, antibiotics and bacteria were imaged and chemically analyzed using confocal fluorescence microscopy and Fourier-transform infrared spectroscopy (FTIR). The interaction between therapies depended on the sequential order of application, metabolic activity, and binding of bacterial cell surface biomolecules. The results demonstrated a potentiating effect of the antibiotic with up to to 32-fold reduction in minimum inhibitory concentrations and mean reductions of 7 log CFU/ml by physicochemical action at bacterial level after the photodynamic treatment. The changes observed as a result of bacteria-antibiotic interactions, such as membrane permeabilization and increase in susceptibility, may be a possibility for solving the problem of microbial multidrug resistance.

## Introduction

According to the World Health Organization (WHO), bacterial infections affect hundreds of millions of people worldwide every year, and some cases result from outbreaks and healthcare-associated infections (HAIs). *Staphylococcus aureus* is one of the main opportunistic human pathogens that cause infections in skin and mucous membranes, endocarditis, pneumonia, and bacteremia under adverse conditions, sometimes leading to fatal conditions^1^. Resistant, tolerant, and antibiotic-resistant bacterial strains can result in failure to treat infections^2^. Antibiotic failures have affected nearly 180 million people worldwide, with a 225 million predicted increase by 2030, predating the overuse of antibiotics for treating patients infected with SARS-CoV-2. Such patients have been wrongly prescribed the drug, considering the co-infection of the virus with the bacteria has been lower than 8%, i.e., the global scenario of bacterial resistance to antibiotics may have been accelerated^3,4^. However, the insertion of new antibiotics in the market has decreased over the years, since new antimicrobials have not generated enough profit for pharmaceutical industries, thus requiring new strategies for the treatment of infections^5,6^ Antibiotic therapy has remained the gold standard for the treatment of infections, despite an increase of antibiotic-resistant and persistent bacteria in chronic cases^7^.

**Figure 1:**
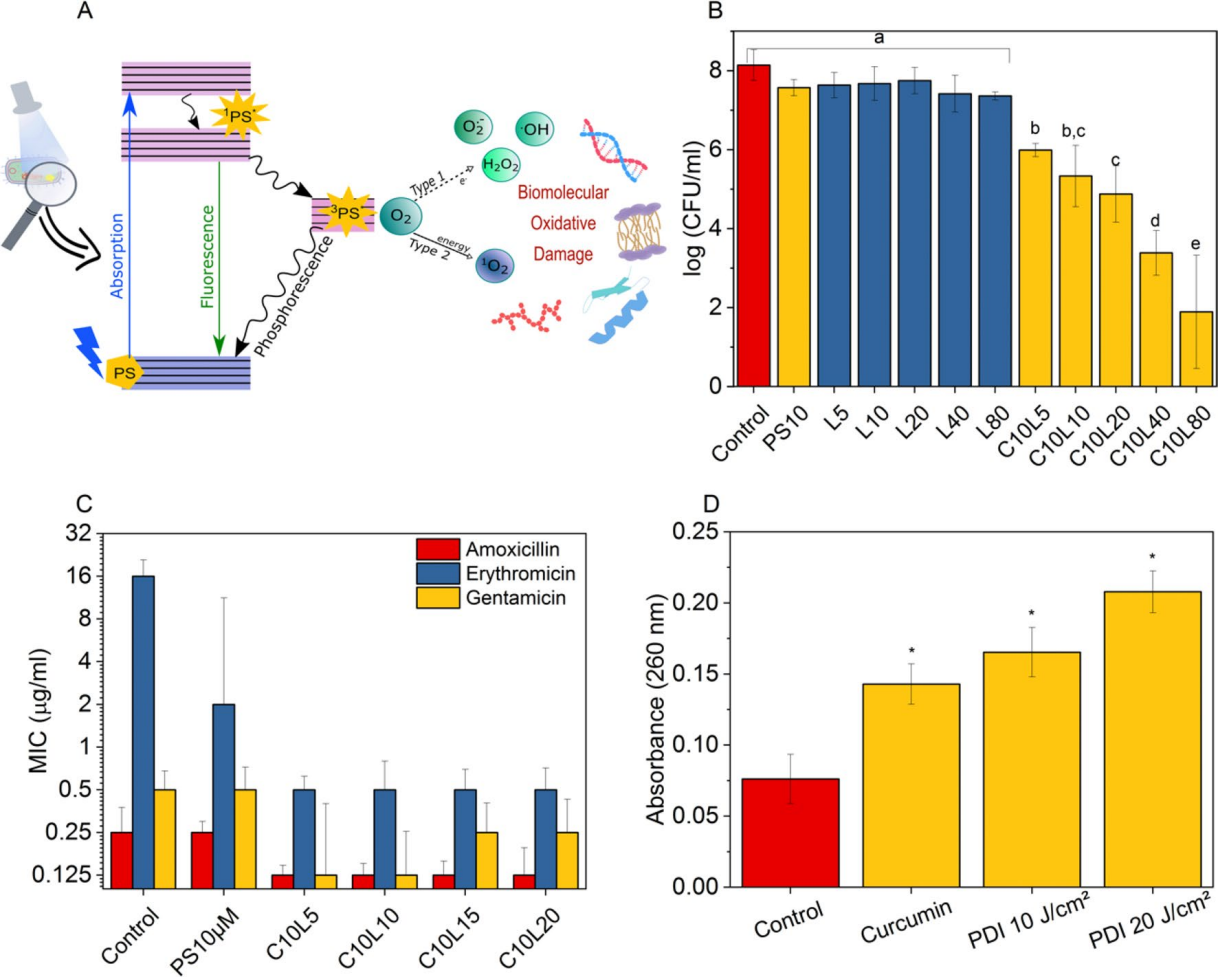
Photodynamic inactivation (PDI) and antibiotic therapy. (**A**) Mechanism of PDI action represented by Jablonski diagram. (**B**) Survival of *S. aureus* with curcumin dark control 10 μM (PS10) curcumin dark control, 5 to 80 J/cm^2^ (L) control light doses, and PDI with fixed PS concentration for different light doses (C10L). Different letters indicate statistically significant difference *p* < 0.05. (**C**) Standard Minimum Inhibitory Concentration (MIC) (control) values for amoxicillin, erythromycin, and gentamicin in combination (or not) with PDI. (**D**) Determination of membrane permeability by the presence of extracellular DNA in bacteria exposed to 10 μM of curcumin in the dark and with a light dose of 10 and 20 J/cm^2^. * indicates statistically significant difference *p* < 0.05.

The combination of treatments may be an alternative to overcome that limitation. Unlike antibiotic therapy, Photodynamic Inactivation (PDI) is based on the specific interaction of a drug with biological targets, as it occurs in bacteria^8^, promoting their death by oxidative stress in multiple cellular structures and preventing the development of resistance^9^. The PDI mechanism is based on the production of reactive oxygen species (ROS) from the absorption of photons by the photosensitizer (PS), promoting an electron from the ground state (S0) to the excited singlet state (S1), with a high probability of transition to the excited triplet state (T1). PS can interact with O2 through two types of reaction, namely I and II^10,11^. In the former, PS in T1 state transfers electrons to organic substrates or biomolecules, forming radical ions that tend to react with molecular oxygen, resulting in ROS such as hydrogen peroxide (H_2_O_2_), superoxide anion radical (O_2_^−^), and hydroxyl (–OH). In contrast, in type II, PS in T1 state transfers energy directly to molecular oxygen through a molecular encounter, exciting it to its highly reactive singlet state (^1^O_2_). All ROS react with proteins, lipids, and nucleic acid, promoting bacterial cell death^5,12^ (see Fig. 1A). A Clinical trial and in vitro studies conducted demonstrated the PDI effectiveness for superficial infections such as pharyngotonsillitis^13^, and internal (e.g., pneumonia)^14^, and prophylactic ones (e.g., endotracheal tubes)^15^. Mutiple studies used curcumin as PS. Curcumin is a molecule extracted from Curcuma longa. It has been approved for use in humans since it is commonly used as a seasoning in cooking. Curcumin is widely known for its pharmacological properties such as anti-inflammatory and antibacterial actions^16^.

Antibiotic therapy and PDI act through a different mechanism towards inactivating bacteria when combined. The ROS presence from PDI may cause synergistic effects during antibiotic treatment, thus potentiating its action^17^. This study evaluated the interaction of curcumin PS with three different conventional antimicrobials, namely amoxicillin (AMO—β-lactam that inhibits cell wall synthesis)^18^, erythromycin (ERY—macrolide), and gentamicin (GEN—aminoglycosides), which inhibit protein synthesis by acting on the 50S and 30S subunits of the ribosome, respectively^19,20^. Moreover, the way proposed for analyses on how different combination protocols affect the interaction between antimicrobial molecules and PS on *Staphylococcus aureus* was observed towards the obtaining of consistent results on the potential of combined therapies for clinical applications. The main long-term goal is to identify alternatives that make antibiotic-resistant bacteria return to the original properties of non-resistance. PDI can be an adjunct to antibiotic therapy, prolonging the use of antibiotics available on the market.

## Methodology

### Micro-organism cultivation

*Staphylococcus aureus* (ATCC 25923) was cultivated in Brain Heart Infusion Agar (BHI) for 24 h at 37 °C, aerobic conditions. Bacteria colonies were suspended in phosphate buffered saline (PBS) for PDI and antibiotic treatments in a Mueller Hinton (MH) medium. The inoculum was adjusted to 10^8^ CFU/ml at 600 nm (Cary UV-Vis50, Varian) and inoculated in confocal dishes for 24 h at 37 °C in a BHI medium for the obtaining of confocal microscopic images.

### Photodynamic inactivation

A stock solution of the PS (5 mM synthetic curcumin (PDTPharma^®^)) was prepared in ethyl alcohol and was diluted in distilled water to concentrations of interest. This way, the concentration of ethyl alcohol was substantially reduced to avoid any potential adverse effects, with final concentration of 0.2%. Three control groups, namely General Control (bacteria), Dark Control (bacteria + PS), and Light Control (bacteria + light) and the PDI treatment group (bacteria + PS + light) were prepared. The bacteria were incubated with 10 μM of curcumin for 15 min for PDI and dark groups. These parameters were chosen based on a pilot study. Subsequently, in 24 well-plates the light and PDI groups were evenly irradiated from the botton with LED lighting device (Biotable^®^—produced by MM Optics—Brazil) at 450 nm wavelength, 40 mW/cm^2^, with a 5 to 80 J/cm^2^ light dose. The light dose determination is given by D = I.t, where “D” is the light dose, “I” the intensity of the irradiation device, “t” the irradiation time. The samples were seeded in Petri dishes for the counting of the surviving colony-forming units per milliliter (CFU/ml). The survival fraction was calculated as indicated by the ${\text{SF}} = \frac{{Log \left( {\frac{CFU}{{ml}}} \right)treated}}{{Log \left( {\frac{CFU}{{ml}}} \right)untreated}}$.

### Minimum inhibitory concentration

The experiments followed the recommendations of the Clinical and Laboratory Standards Institute (CLSI)^21^. Antimicrobial agents amoxicillin (AMO), erythromycin (ERY), and gentamicin (GEN) were distributed in 96-well plates by sequential dilution and the bacterial inoculum with standardized concentration was subsequently added. The final bacterial concentration was approximately 10^6^ CFU/ml. A positive control of bacterial growth and a negative control of the culture medium were performed together and the plate was stored at 37 °C for 24 h. A resazurin solution (0.002%) was then added and kept for 4 h at 37 °C. The minimum inhibitory concentration (MIC) was defined as the lowest concentration of the antibiotic capable of inhibiting the visible growth of bacterial strains^21^. In the MIC study after PDI, the added inoculum corresponds to the bacteria treated according to the protocol described in the previous (“Photodynamic inactivation” section). First, the curcumin is internalized by the bacteria for 15 min at 37 °C, then the sample is irradiated in the 24-well plate. It is then transferred to the 96-well plate for the MIC experiment. PDI sublethal conditions were used for the combination experiments, ensuring bacterial cell survival.

### Combination of photodynamic inactivation and antibiotic therapy

#### Oxidative stress

The first treatment applied to *S.aureus* followed the protocol described in “Photodynamic inactivation” section. The bacteria were incubated in the presence of 10 μM of curcumin for 15 min and subsequently irradiated at 10 and 20 J/cm^2^. Surviving bacterial cells were added to a MH medium containing AMO, ERY, and GEN at different concentrations (1/4 MIC, ½ MIC, MIC, 2 MIC, 4 MIC) for 24 h at 37 °C. The samples were then diluted and plated on a BHI agar medium for colony counting after 24 h.

#### Simultaneous internalization

The bacterial inoculum was incubated at 37 °C in the presence of 10 μM of curcumin and different concentrations of antibiotics (from 1/4 MIC to 4 MIC) in MH for 15 min. It was subsequently irradiated at 10 and 20 J/cm^2^ and, after 24 h-incubation at 37 °C, diluted and plated on a BHI agar medium for colony counting after 24 h.

### Permeability of the cytoplasm membrane

The protocol for the evaluation of the cytoplasmic membrane permeabilization by measurements of the extracellular genetic material absorbance was adapted from Siriwon et al.^22^. *S.aureus* bacterium was subjected to the PDI treatment described elsewhere (2.2). After treatment, the samples were collected and filtered through a sterile nitrate cellulose membrane (0.22 µm) and the absorbance of the supernatant was measured at 260 nm (Cary UV-Vis50, Varian).

### Treatment cycle

The following two protocols were applied for cyclic treatments combining PDI and ANTB:

#### Concomitant cycle

The bacteria were incubated with 10 μM curcumin for 15 min and subsequently irradiated with 10 J/cm^2^ at 450 nm by Biotable^®^. The cells were then suspended in MH containing AMO, ERY, and GEN separately under previously established MIC conditions and incubated at 37 °C for 6 hours^23^. Subsequently, they were washed and samples were collected for plating and determination of CFU/ml. The washed cells were subjected to the same protocol for an additional 5 cycles.

#### Switching cycle

At time 0 h, the bacteria were incubated with 10 μM curcumin for 15 min and then irradiated with 10 J/cm^2^ at 450 nm by Biotable^®^. The surviving cells were suspended in only MH, incubated at 37 °C for 6 h, and subsequently washed. Samples were collected for plating and determination of CFU/ml. The washed cells were suspended in MH containing AMO, ERY, and GEN antibiotics separately under previously established MIC conditions and incubated at 37 °C for 6 h. The procedure was repeated for 30 h. At times 0, 12, and 24 h, *S. aureus* received PDI and at 6 and 18 h, an antibiotic therapy was administered.

### Fourier transformation infrared spectroscopy

Colonies from the plated samples from the treatment cycle (2.6) at 37 °C for 24 h were collected for analysis by Attenuated Total Reflection (ATR) on the Agilent Cary 630 FTIR Spectrometer^®^ instrument. Colonies were evenly distributed over the crystal surface. A dry sample was scanned 250 times and the result was the average of the measurements. The FTIR spectrum was measured in the 4000 to 650 cm^−1^ range in three different samples. The spectrum of bacteria from each treatment was processed according to the steps: (1) calculation of the second derivative, (2) normalization by minimum–maximum and (3) analysis of hierarchical clustering by Origin^24^.

### Confocal microscopy analysis

An inverted Zeiss LSM 780 confocal laser scanning microscope (CLSM) with a Coherent Chameleon laser (Ti:Sapphire) as a two-photon (2P) excitation source tuned to an 800 nm wavelength was used in the experiments with 80 MHz laser pulse. Images were collected in both spectral and channel modes with an objective lens (63× , 1.2 numerical aperture, water immersion). Planktonic bacterial cells were conditioned in sterilized confocal dishes and maintained at 37 °C at 7% CO_2_. The final measurement water volume for each well was 200 μl. The cell viability was measured with the use of LIVE/DEAD™ reagent with 2-photon (2P) excitation at 800 nm in channel mode and fluorescence was captured between 415 and 540 nm for acridine orange and 580 to 620 nm for ethidium bromide. The experiments were conducted when viability exceeded 90%. The optical setup was adjusted to the best signal-to-noise ratio and fixed when different samples were compared.

#### *Fluorescence lifetime imaging (FLIM*)

2P laser was pulsed at 80 MHz for FLIM experiments and a beam splitter divided fluorescence into two detecting channels of a PicoQuant GmbH system that detected it between 500 and 550 nm. Time correlated single photon counting (TCSPC) with an avalanche detector of time response limited at approximately 100 ps was the method adopted. A two-exponential fit adjusted the fluorescence decay data and Time Trace Analysis by PicoQuant GmbH software chose the fitting range considering the decay part of the time-dependent data according to optimal parameters.

#### Photosensitizer uptake

The average laser power was adjusted between 1.1 and 4% so that the fluence of emission photons per pixel would be sufficient for image collection in channel and spectral mode. The scan was adjusted for image collection every 30 s for 30 min with low dose (< 0.5 J/cm^2^) with no measurable photodegradation damage.

#### Fluorescence recovery after photodegradation (FRAP)

The samples were photodegraded in a specific rectangular region of the image whose focal plane was always set at 10 µm above the glass slide interface. The power of the two-photon laser at 800 nm in the photodegraded region was set to 40% of the nominal intensity (300 microwatts), whereas 2% of the power were used for scanning the entire image. The photodegradation process started after the collection of 5 images (7.75 s/image). Fifteen recovery images were collected and the emission detection wavelength ranged from 490 to 585 nm.

### Statistical analysis

The experiments were performed in independent triplicates (N = 9) for each group studied. Shapiro–Wilk test evaluated the normal distribution of the data. The results were analyzed by ANOVA associated with post hoc Tukey test. The error bar was determined by standard deviation. The *p*-value < 0.05 was considered statistically significant. Bliss independence model^25^ analyzed the synergistic and antagonistic effects of the combination of therapies according to

$${\text{S}}_{{{\text{bliss}}}} = {\text{SF}}_{{{\text{antb}}}} *{\text{SF}}_{{{\text{PDI}}}} - {\text{SF}}_{{{\text{Comb}}}}$$

## Results

### Photodynamic and antibiotic inactivation

Figure 1B shows the survival of *S. aureus* in PDI as a function of light dose and fixed concentration of curcumin. No microbial toxicity was observed with the curcumin use in the dark—only with a light application. The 10 μM curcumin condition with 10 and 20 J/cm^2^ light doses was evaluated in combination therapies, resulting in 2.81 and 3.27 log (CFU/ml) bacterial death, respectively.

Amoxicillin (β-lactam), erythromycin (macrolide), and gentamicin (aminoglycoside) showed MIC, indicating the studied strain is sensitive to antibiotics—the exception was erythromycin, classified as resistant. As observed by the different MICs, antibiotic therapy has a specificity that depends on the antibiotic used, whereas the conditions of inactivation by PDI are constant. Previous PDI application potentiated the action of the three antibiotics. Surviving microbial cells were more sensitive, achieving a 2 to 32-fold reduction in MICs. Bacterial exposure to curcumin in the dark did not change the initial MIC values for AMO and GEN, and an eightfold reduction was observed in the number of bacterial cells treated with ERY. In general, the reductions in MIC values promoted by the PDI previous application remained constant, regardless of the dose of light applied, showing synergistic combinations can be identified according to the treatment conditions and the class of antibiotic chosen.

The presence of extracellular DNA was quantified for assessments of the damage caused to the bacterial plasma membrane by PDI. Figure 1D indicates the existence of genetic material of PDI groups in relation to the control group. The highest light dose (20 J/cm^2^) applied to PDI showed a greater presence of extracellular DNA in the supernatant, indicating bacterial membrane damage due to the increase in its permeability.

### Effect of combination of therapy protocols

**Figure 2:**
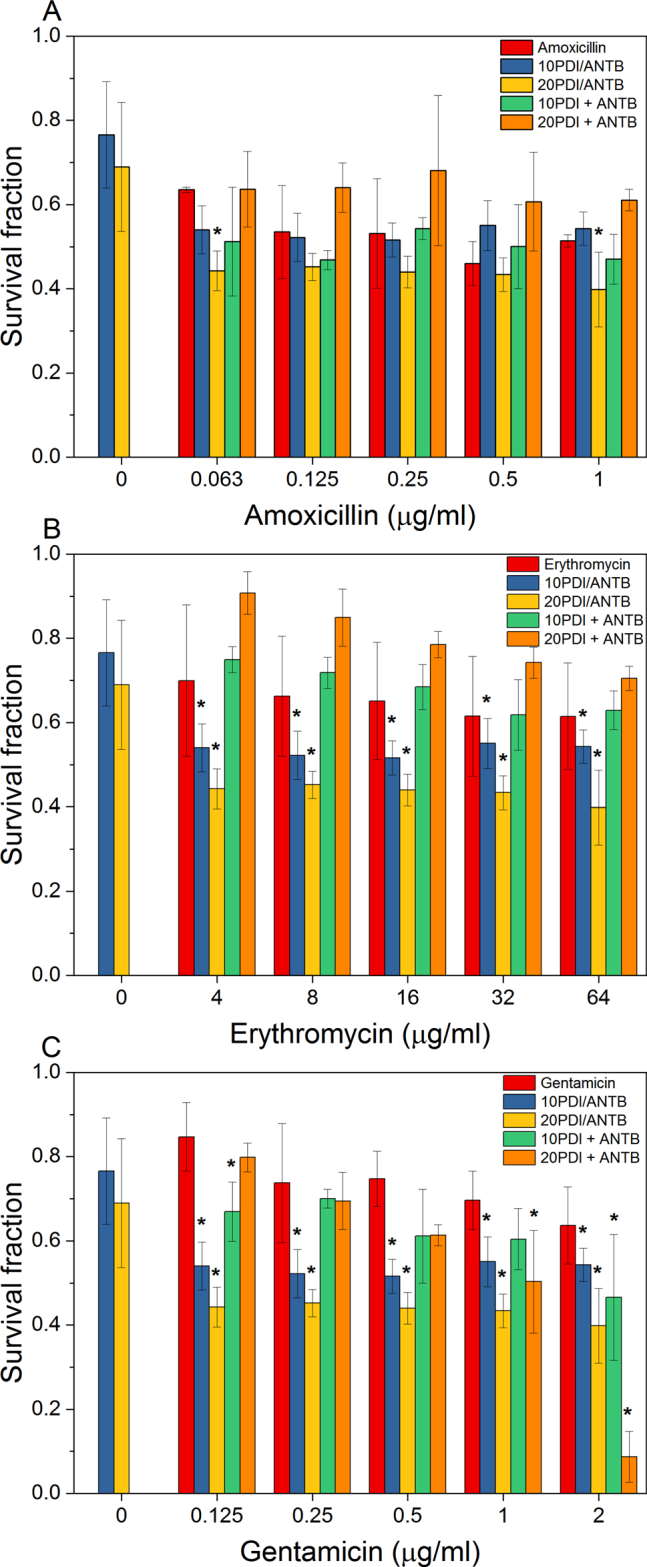
Survival curve of combined treatments by oxidative stress (PDI/ANTB) and Simultaneous internalization (PDI + ANTB) with application of 10 μM of curcumin and 10 and 20 J/cm^2^. (**A**) Amoxicillin, (**B**) Erythromycin, (**C**) Gentamycin. Normalizations performed for untreated bacteria. *indicates synergistic combination for Bliss independence.

The order influence of different combinations of PDI protocols with antibiotic therapy on inactivation responses was evaluated. Figure 2 displays the survival response of *S. aureus* at different antibiotic concentrations close to the MIC value. Non-normalizing values of log CFU/ml are shown in Table S1. The bacteria first received PDI and then the antibiotic in the group oxidative stress. All combined groups of ERY and GEN with PDI showed a synergistic response, i.e., a lower proportion of bacterial cell survival compared to monotherapies (ANTB or PDI only). The groups treated with AMO combined with PDI (20 J/cm^2^) had a lower survival fraction than the respective monotherapy groups; therefore, only a few groups were synergistic. Moreover, the microbial survival was higher compared to antibiotic therapy, but lower than PDI monotherapy (10 J/cm^2^) at high AMO concentrations. In terms of Log (CFU/ml) reduction, the maximum obtained for the oxidative stress group (PDI/ANTB) was 5.65 Log (CFU/ml) to PDI_20_/AMO_1_ and 9.5 Log (CFU/ml) to PDI_20_/ERY_64_ and PDI_20_/GEN_>0.25._

According to the simultaneous internalization protocol, ANTB is irradiated with PS, i.e., PS and ANTB are internalized simultaneously in the previously untreated cells. Most results were antagonistic for AMO and ERY, with a higher survival in comparison to the monotherapies. GEN provided both synergistic and antagonistic results, mainly as a function of the antibiotic concentration used—with increasing concentration being inversely proportional to microbial survival for both light doses applied. In terms of Log (CFU/ml) reduction, the maximum obtained for the internalized simultaneously (PDI + ANTB) was 5.08 Log (CFU/ml) to PDI_10_ + AMO_0.125_ and 3.65 Log (CFU/ml) to PDI_10_ + ERY_32_ and PDI_20_ + GEN_2._

In general, antibiotic irradiation in the simultaneous internalization protocol leads to mostly antagonistic results, due to smaller reductions in bacterial mortality from the interaction between curcumin and antibiotics for reducing availability for antimicrobial action. The results show not only the concentrations of antibiotics, but also PS and the light dose influence the achievement of synergistic results. However, the protocol applied can be decisive.

### Dynamics of bacteria interaction with photosensitizers and antibiotics

**Figure 3:**
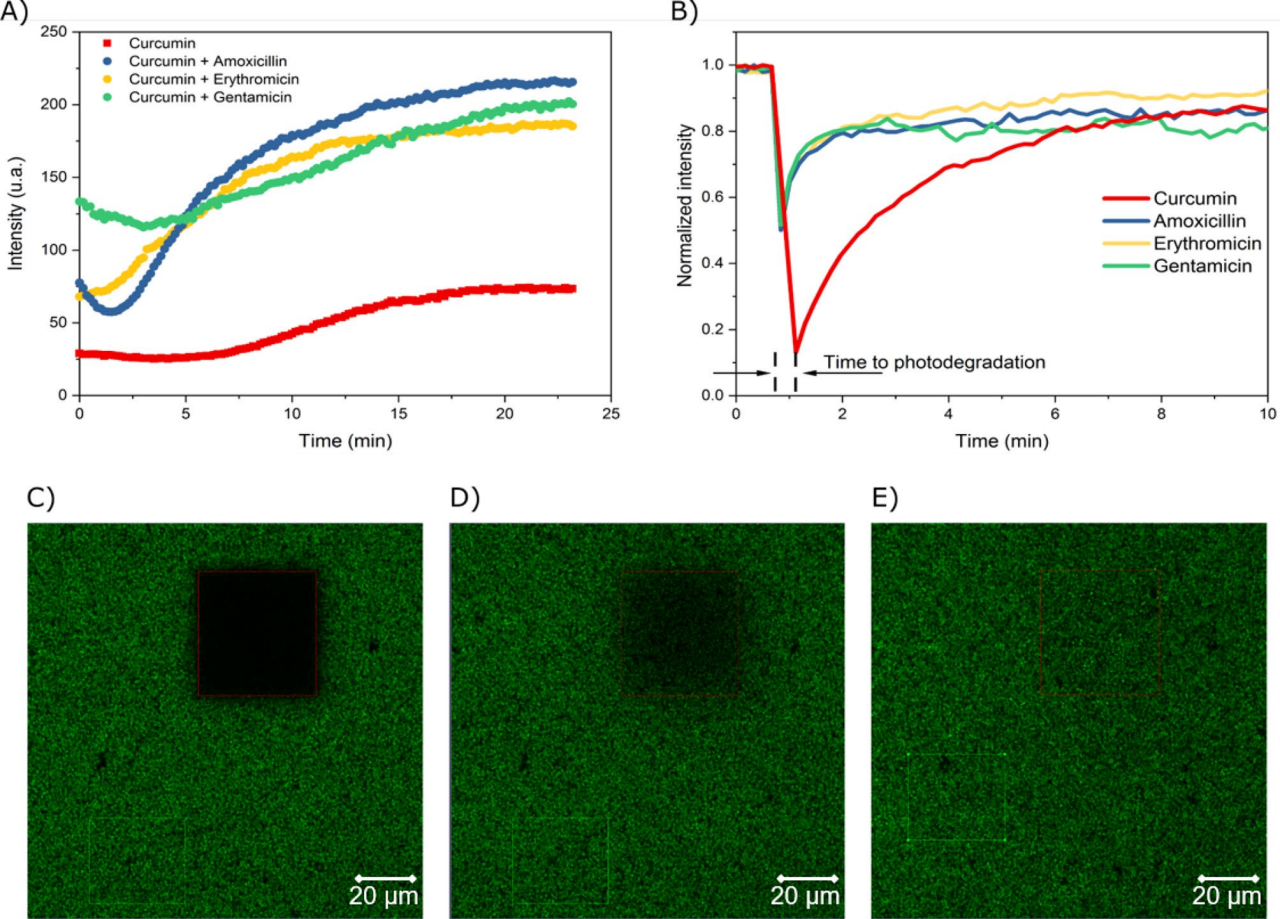
Confocal microscopy data of 10 μM curcumin fluorescence in *S. aureus* and in the presence of amoxicillin, erythromycin, and gentamicin. (**A**) Kinetics of curcumin incorporation. (**B**) Fluorescence Recovery After Photodegradation (FRAP) of curcumin. Image of photodegradation of internalized curcumin with 63 × magnification and channel mode with (**C**) 1min7s D) 2min7s and (**E**) 10min1s timelapse.

The results of mostly antagonistic simultaneous curcumin internalization with the antibiotic followed by irradiation were investigated by confocal fluorescence microscopy. Figure 3A shows the kinetics of incorporation of curcumin alone and in the presence of AMO, ERY and GEN. Comparatively, the kinetic behavior of curcumin alone is distinct from that in presence and absence of antibiotics. The initial concentration incorporated (t = 0 min) is 40% lower than the final one; however, its increase reaches up to 2.5-fold, a proportion similar to that in the presence of antibiotics. Bacteria with CUR and ERY show an immediate internalization dynamic. However, CUR only and in the presence of AMO and GEN has a minimum time to start its active incorporation by the bacteria. Showing us, therefore, indications of disturbance in the control of membrane permeability by reducing the fluorescence intensity of the internalized PS. Interestingly, due to experimental limitations, there is an initial 30 s average interval for fluorescence collection, which was the same for all groups in all repetitions.

Figure 3B shows the photodegradation action of curcumin incorporated by the bacteria is more pronounced for curcumin alone, with a 1.75-fold decrease in fluorescence intensity than in the presence of antibiotic (Figure S1). This effect is related to the photodegradation of PS, thus resulting in the production of ROS, which is more prominent for curcumin alone (Fig. 3C–E). The recovery time of internalized curcumin, with a maximum 85% in 2.1 min, was slower than in the presence of antibiotics, observed at an average recovery rate of approximately 70% in 0.4 min, which facilitated the internalization of the PS, but not necessarily the PDI. As expected, it was faster due to the presence of antibiotics, which facilitates the PS internalization, but does not necessarily favor PDI. The low photodegradation of curcumin in the presence of antibiotics may be related to the decrease in ROS production, which is in agreement with the results in Fig. 2, considering the simultaneous internalization protocol exerts a lower antimicrobial effect.

### Bacterial response to treatment cycle

**Figure 4:**
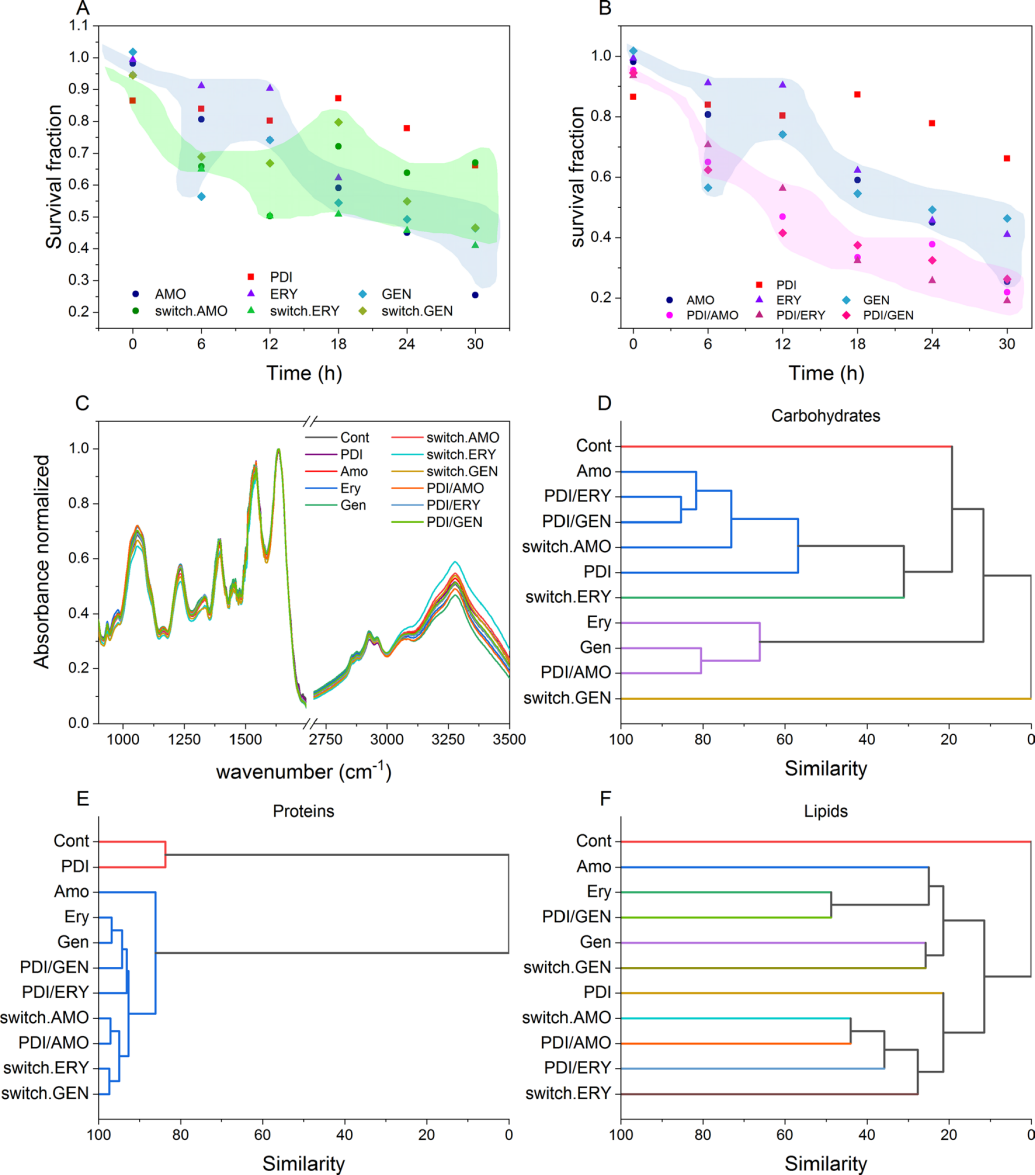
Treatment cycle. Red: PDI with 10 μM of curcumin and 10 J/cm^2^ and Blue: antibiotic group. Normalizations performed for untreated bacteria. (**A**) Combined treatment switched every 6 h, starting with PDI (switch.ANTB). (**B**) Combined treatment—concomitant PDI and ANTB every 6 h (PDI/ANTB). (**C**) FTIR absorbance spectrum of surviving bacteria after 12 h of treatment cycle of PDI, AMO, ERY, and GEN monotherapies; sequentially combined treatments every 6 h (PDI/ANTB), and switching PDI and antibiotics every 6 h (switch.ANTB). Hierarchical Cluster Analysis of the 2nd derivative of the FTIR absorption spectrum of regions (**D**) 900–1200 cm^−1^, corresponding to carbohydrates, (**E**) 1500–1800 cm^−1^, corresponding to protein, and (**F**) 2800–3100 cm^−1^, corresponding to fatty acids. Colors of the dendrogram indicate clustering of similarity greater than 50%. A statistically significant difference was considered *p* < 0.05.

In clinical practice, ANTBs are administered periodically, therefore combined treatment cycles for 30 h were simulated (Fig. 4). The survival result of PDI monotherapy in the first hours was equivalent to that of ANTB; however, the PDI response does not change considerably over time, with the survival fraction varying from 0.86 to 0.66, whereas the survival of the AMO, ERY and GEN declines from 0.98 to 0.25, 0.99 to 0.41, and 1.02 to 0.46, respectively. Figure 4A shows the survival of *S. aureus* in a combined treatment consisted in switching PDI and antibiotic therapy (switch.ANTB) every 6 h. In the first hours, the bacterial cells were weaker, due to the fast action of PDI, favoring the action of ANTB and showing a lower survival when compared to monotherapies. However, the bacteria treated with switch therapies showed a greater survival over time when compared to antibiotics monotherapies, i.e., the switching of treatments for long periods is more effective than PDI monotherapy, although antibiotic therapy is superior to those two protocols.

Figure 4B displays the bacteria treated with the combination of PDI and ANTB every 6 h by the oxidative stress protocol, which proved more effective (PDI/ANTB). The result shows the combination of therapies significantly reduces the survival of bacteria compared to monotherapies (*p*-value < 0.05), since the PDI action is immediate in bacterial cells and the action of ANTBs is effective over time. Concomitant cycle has stood out as a better protocol—as an example, at 12 h, PDI monotherapy has a reduction in bacterial survival of 20% and for ANTB an average of 21%, but for the concomitant cycle the reduction is on average 52%, which indicates a synergistic and not an additive effect.

Colonies surviving from treatment cycles (12 h) were evaluated by FTIR spectroscopy to consider the effects of monotherapies and their combinations on the vibrational bonds of biomolecules present on the bacterial cell surface (Fig. 4C). By having molecules bound to a membrane and the cell wall, either constituted or by adhesion, their modes of interaction with light can be affected due to modifications caused by neighboring molecules. The observation of changes in the frequencies at which the molecules interact with radiation, exciting their vibrational modes, can give indications of the local conditions where the molecule is found. Membrane integrity, proteins, wall composition can thus be investigated. The 900–1200 cm^−1^ region corresponds to carbohydrates^26^, and, as shown in Fig. 4D. The analyzed treatments were arranged into five groups, whose similarity is greater than 50%. The control remained at 20% similarity with 6 treatments (Fig. 4D blue) and maintained no degree of similarity with PDI/GEN treatment. Regarding proteins (1500–1800 cm^−1^)^24^, the control and PDI maintained an 83.8% similarity, whereas the proteins are totally different from the other treatments for both groups, despite their 86.2% similarity. The analysis of significance in relation to similarity in the second group (Fig. 4E blue) ERY being greater than AMO is related to the mechanisms of antibiotics action on bacterial proteins. According to Fig. 4F, no treatment shares similarity with the control in relation to fatty acids (2800–3100 cm^−1^)^26^. The similarity between the treatments was only 64.7% (Fig. 4F blue), except for PDI, which showed a 45.6% similarity. The analysis of changes in the vibrational modes of lipids is consistent with the results in Fig. 3. In general, the application of any treatment, whether combined or monotherapy, affected the vibrational states of bacterial surface biomolecules.

## Discussion

The increase in the number of bacterial strains resistant and persistent to existing antimicrobials over the years has motivated studies to enhance the action of antibiotics in microbial control^27^. Antibiotic-resistant bacteria modify drug binding sites, increasing the expression of efflux pumps or enzymes that degrade such molecules, which is of particular interest for combined therapies that act in another action mechanism and, in certain situations, may potentiate the drug action. Antibiotic effectiveness does not correspond to only the ligand-receptor interaction, as they also promote other metabolic responses^28^, such as increase in basal production of ROS for some bactericidal antibiotics of the β-lactam class and aminoglycosides^17,29,30^. Oxidative stress can be potentiated with the introduction of PDI in a combination treatment.

The application of PDI prior to that of antibiotic under sub-inhibitory conditions causes damage to the membrane, affecting its permeability. Curcumin shows predisposition to interact with the lipid bilayer and influences the dynamics of gramicidin channels^31^. The increase in membrane permeability may favor the internalization of the antibiotic, which is a prerequisite for its effectiveness^2^, one of the reasons that prior application of curcumin resulted in increased susceptibility to ERY (Fig. 1C). Moreover, the production of ROS promoted by the interaction of curcumin with blue light affects not only the membrane, but also the various cellular components, weakening or eliminating bacterial cells^16^. The ROS produced can act on lipid peroxidation that compromises the structural integrity and increases the permeability of Na^+^ and K^−^ ions, inhibit DNA and RNA replication by oxidizing the sugars of these biomolecules, degrade proteins and enzymes by damage caused to tryptophan, methionine and lysine residues^11,12,32^. A comparison between the protocols evidenced the previous damage caused by sub PDI to both membrane and cellular metabolic processes weakens the bacteria, making them more susceptible to other antimicrobials. Therefore, a synergistic effect in the oxidative stress protocol was observed decreasing MIC values, as also demonstrated in other planktonic cultures^2,33,34^ and biofilms^35,36^.

Synergistic or antagonistic responses are dependent on the different parameters of each monotherapy applied. Although both antimicrobial techniques are being combined, the mechanisms of action do not occur simultaneously, but sequentially. In this study, a simultaneous internalization of curcumin with AMO, ERY, and GEN was less efficient in bacterial inactivation than the oxidative stress protocol. Although the irradiated wavelength is not absorbed by antibiotics, we assume such molecules, when irradiated, can promote disturbances in the medium. Antibiotics can act together with biomolecules as ROS targets for reduce the effectiveness of PDI (Figure S2, S3).

OH, O_2_^−^, ^1^O_2_ from advanced oxidation processes (AOPs) such Fenton oxidation, photocatalytic oxidation, and electrochemical oxidation, have promoted oxidation and reduction of several classes of antibiotics in drinking water treatments, leading to compounds with preserved central structures and intermediates as reaction products^37,38^. Similarly, the presence of the antibiotic during the production of ROS by PDI corresponds to the same environment of AOPs, i.e., the antibiotic degrades during the photodynamic action.

The efficiency of a PS can be modulated with the environment in which it is present. Regarding curcumin, since it is a mostly hydrophobic molecule, the molecules are expected to be aggregated in an aqueous environment, as evidenced by the predominance of short lifetimes and the red shift in the emission spectrum also in the presence of the antibiotic in an aqueous medium (Figure S2). PS molecules uptaken are free inside the cell. The decay is different from a monoexponential one, showing higher efficiency in the production of ROS when compared to external molecules in solution. However, because the intracellular concentration is approximately in the millimolar order of magnitude, we have hypothesized curcumin molecules are spatially distributed close to the energy transfer radius (Eq. S1-S3). Therefore, when a photon is absorbed, the efficiency of energy transferred to oxygen molecules is lower due to increased non-radiative transfers between curcumins (Figure S3), thus decreasing the efficiency of PDI when compared to the oxidative stress protocol, in which the absence of antibiotics reduces the concentration of internalized curcumin.

The time of curcumin fluorescence decay inside the microorganism is severely affected by both concentration conditions and presence of antibiotics. The decay is different from a monoexponential, as indicated by the nonlining of fluorescence curves. Such a situation goes beyond the excited state of PS changes. Initially, the non-saturated case always decreases more, demonstrating the saturation regime of the concentration leads to a complex state of change in the environment for the curcumin molecule. Because the behavior is related to the antibiotic, such molecules should have a fundamental participation in the process.

To better understand the behavior the S2 and S3 figure shows all the data observed. From the point of view of influencing the lifetime of the excited state of curcumin, it is observed that AMO is lower than the ERY, which in turn is less than GEN. This also demonstrates that this should be the order of curcumin interaction with the antibiotic molecules, that is GEN > ERY > AMO.

Differently from antibiotic therapy, PDI does not promote the selection of resistant bacteria even if the sub-inhibitory conditions of the treatments are applied^39,40^. The limitations of PDI result in low efficacy for infection treatment, mainly due to the PS incorporation and light penetration into infectious biological tissues. Any technique offers advantages and disadvantages; therefore, when combining therapies, in addition to aiming at potentiated antimicrobial results, one should seek to minimize the disadvantages, as is the case with PDI and antibiotic therapy. Furthermore, not only the inhibitory effects should be analyzed during the combination of treatments, but also the effects on the surviving cell structures and their metabolic activity must be observed.

According to the results of the present study, combined treatments led to greater efficiency responses than the monotherapies. The combined use of PDI with different classes of antibiotics can result in different antimicrobial performances due to bacterial incorporation rate and vibrational states of biomolecules.

## Conclusions

PDI can potentiate the action of ANTB when both are combined. Antibiotics act from the internalization of the molecules into the cell, since the target-actions of such drugs are in intracellular structures. The MIC reduction with previous PDI application demonstrated the cell damage, mainly in the membrane, affects cell permeability, favoring the antimicrobials uptake for increasing the antibiotic susceptibility of bacteria, These results are promising in the perspective of reversing the scenario of resistant and persistent microorganisms.

Different combination protocols of both therapies exert synergistic or antagonistic effects. The ROS produced by PDI can weaken the cell, facilitating the action of antibiotics, or degrade the antimicrobials, decreasing their efficiency. Moreover, the antibiotic and photosensitizer interaction may compromise photodynamic efficiency. The previous application of PDI has stood out as the best temporal sequence of combination of the studied therapies. Although the interaction between therapies may not lead to trivial results, the adjustment of the parameters enhances the antimicrobial action in both sensitive and resistant strains, which may be a solution to antibiotic failures.

## Supplementary Information

Supplementary Information 1.

Supplementary Information 2.

## Data Availability

The datasets used and/or analyzed are available from the corresponding author on reasonable request. Supplementary information contains FTIR spectroscopy data for *S. aureus* with PDI and antibiotic treatments.

## References

[1] Rowe SE (2020). Reactive oxygen species induce antibiotic tolerance during systemic *Staphylococcus aureus* infection. Nat. Microbiol..

[2] Song M (2020). A broad-spectrum antibiotic adjuvant reverses multidrug-resistant gram-negative pathogens. Nat. Microbiol..

[3] Lansbury L, Lim B, Baskaran V, Lim WS (2020). Co-infections in people with COVID-19: A systematic review and meta-analysis. J. Infect..

[4] Langford BJ (2021). Antibiotic prescribing in patients with COVID-19: Rapid review and meta-analysis. Clin. Microbiol. Infect..

[5] Maisch T (2009). A new strategy to destroy antibiotic resistant microorganisms: Antimicrobial photodynamic treatment. Mini-Rev. Med. Chem..

[6] Yadav S (2020). Making of water soluble curcumin to potentiate conventional antimicrobials by inducing apoptosis-like phenomena among drug-resistant bacteria. Sci. Rep..

[7] Blair JMA, Webber MA, Baylay AJ, Ogbolu DO, Piddock LJV (2015). Molecular mechanisms of antibiotic resistance. Nat. Rev. Microbiol..

[8] Peterson E, Kaur P (2018). Antibiotic resistance mechanisms in bacteria: Relationships between resistance determinants of antibiotic producers, environmental bacteria, and clinical pathogens. Front. Microbiol..

[9] Wozniak A, Grinholc M (2018). Combined antimicrobial activity of photodynamic inactivation and antimicrobials-state of the art. Front. Microbiol..

[10] Wainwright M (2017). Photoantimicrobials—Are we afraid of the light?. Lancet Infect. Dis..

[11] Bacellar I, Tsubone T, Pavani C, Baptista M (2015). Photodynamic efficiency: From molecular photochemistry to cell death. Int. J. Mol. Sci..

[12] Hamblin MR, Hasan T (2004). Photodynamic therapy: A new antimicrobial approach to infectious disease?. Photochem. Photobiol. Sci..

[13] Blanco KC (2018). PDT, an adjuvant therapy to antibiotic failure in streptococcal tonsillopharyngitis. Biomed. J. Sci. Tech. Res..

[14] Kassab G (2020). Safety and delivery efficiency of a photodynamic treatment of the lungs using indocyanine green and extracorporeal near infrared illumination. J. Biophotonics.

[15] Zangirolami AC, Inada NM, Bagnato VS, Blanco KC (2018). Biofilm destruction on endotracheal tubes by photodynamic inactivation. Infect. Disord. Drug Targets.

[16] Dias LD, Blanco KC, Mfouo-Tynga IS, Inada NM, Bagnato VS (2020). Curcumin as a photosensitizer: From molecular structure to recent advances in antimicrobial photodynamic therapy. J. Photochem. Photobiol. C.

[17] Brynildsen MP, Winkler JA, Spina CS, MacDonald IC, Collins JJ (2013). Potentiating antibacterial activity by predictably enhancing endogenous microbial ROS production. Nat. Biotechnol..

[18] Aronson, J. K. Penicillins. *Meyler’s Side Effects of Drugs* 591–611. 10.1016/B978-0-444-53717-1.01237-3 (Elsevier, 2016).

[19] Majer J (1978). Macrolide antibiotics. J. Chromatogr. Libr..

[20] Aronson, J. K. Aminoglycoside antibiotics. In *Meyler’s Side Effects of Drugs* 216–236. 10.1016/B978-0-444-53717-1.00270-5 (Elsevier, 2016).

[21] Clinical and Laboratory Standards Institute. *Performance Standards for Antimicrobial Susceptibility Testing* 27th ed. CLSI supplement M100. (Clinical and Laboratory Standards Institute, Wayne, PA, 2017).

[22] Siriwong S, Teethaisong Y, Thumanu K, Dunkhunthod B, Eumkeb G (2016). The synergy and mode of action of quercetin plus amoxicillin against amoxicillin-resistant Staphylococcus epidermidis. BMC Pharmacol. Toxicol..

[23] Fridman O, Goldberg A, Ronin I, Shoresh N, Balaban NQ (2014). Optimization of lag time underlies antibiotic tolerance in evolved bacterial populations. Nature.

[24] Lasch, P. & Naumann, D. Infrared spectroscopy in microbiology. in *Encyclopedia of Analytical Chemistry* 1–32 (John Wiley & Sons, Ltd, 2015). 10.1002/9780470027318.a0117.pub2.

[25] Courtney CM (2017). Potentiating antibiotics in drug-resistant clinical isolates via stimuli-activated superoxide generation. Sci. Adv..

[26] Faghihzadeh F, Anaya NM, Schifman LA, Oyanedel-Craver V (2016). Fourier transform infrared spectroscopy to assess molecular-level changes in microorganisms exposed to nanoparticles. Nanotechnol. Environ. Eng..

[27] Pontes MH, Groisman EA (2019). Slow growth dictates non-heritable antibiotic resistance in Salmonella enterica. Sci. Signal..

[28] Zou L (2018). Synergistic antibacterial activity of silver with antibiotics correlating with the upregulation of the ROS production. Sci. Rep..

[29] Vatansever F (2013). Antimicrobial strategies centered around reactive oxygen species—bactericidal antibiotics, photodynamic therapy, and beyond. FEMS Microbiol. Rev..

[30] Takahashi N (2017). Lethality of MalE-LacZ hybrid protein shares mechanistic attributes with oxidative component of antibiotic lethality. Proc. Natl. Acad. Sci. USA.

[31] Ingolfsson HI, Koeppe RE, Andersen OS (2007). Curcumin is a modulator of bilayer material properties. Biochemistry.

[32] Wainwright M (2003). Local treatment of viral disease using photodynamic therapy. Int. J. Antimicrob. Agents.

[33] Aroso RT (2022). Synergic dual phototherapy: Cationic imidazolyl photosensitizers and ciprofloxacin for eradication of in vitro and in vivo E. coli infections. J. Photochem. Photobiol. B.

[34] Willis JA (2022). Breaking down antibiotic resistance in methicillin-resistant *Staphylococcus aureus*: Combining antimicrobial photodynamic and antibiotic treatments. Proc. Natl. Acad. Sci..

[35] Ronqui MR, De AguiarColetti TMSF, De Freitas LM, Miranda ET, Fontana CR (2016). Synergistic antimicrobial effect of photodynamic therapy and ciprofloxacin. J. Photochem. Photobiol. B.

[36] Barra F (2015). Photodynamic and antibiotic therapy in combination to fight biofilms and resistant surface bacterial infections. Int. J. Mol. Sci..

[37] Ge L (2019). The importance of reactive oxygen species on the aqueous phototransformation of sulfonamide antibiotics: Kinetics, pathways, and comparisons with direct photolysis. Water Res..

[38] Jeong J, Song W, Cooper WJ, Jung J, Greaves J (2010). Degradation of tetracycline antibiotics: Mechanisms and kinetic studies for advanced oxidation/reduction processes. Chemosphere.

[39] Soares JM, Inada NM, Bagnato VS, Blanco KC (2020). Evolution of surviving Streptoccocus pyogenes from pharyngotonsillitis patients submit to multiple cycles of antimicrobial photodynamic therapy. J. Photochem. Photobiol. B.

[40] Kashef N, Hamblin MR (2017). Can microbial cells develop resistance to oxidative stress in antimicrobial photodynamic inactivation?. Drug Resist. Updates.

