# Supplementary material for: Physicochemical mechanisms of bacterial response in the photodynamic potentiation of antibiotic effects
Source: Sci Rep. 2022 Dec 7;12:21146. doi: 10.1038/s41598-022-25546-y (PMC9729225; doi:10.1038/s41598-022-25546-y)
Supplement: Supplementary file 2 — Supplementary Information 2. [file 41598_2022_25546_MOESM2_ESM.docx]

**Supplementary Information for**

**Physicochemical mechanisms of bacterial response in the photodynamic potentiation of antibiotic effects**

Jennifer M. Soares^1,^*, Francisco E. G. Guimarães^1^, Vladislav V. Yakovlev²,

Vanderlei S. Bagnato^1,2^, and Kate C. Blanco^1,2^.

^1^ São Carlos Institute of Physics, University of São Paulo – São Carlos - SP, Brazil

^2^ Biomedical Engineering, Texas A&M University - College Station - TX, USA

*Corresponding author: Av. Trabalhador São-carlense, 400, São Carlos, São Paulo, Brazil,

Postal code: 13566-590, +55 (16) 3373-9810, Fax: 3373-9811. Email: [jennifer.soares@usp.br](mailto:jennifer.soares@usp.br)

**This PDF file includes:**

**Page**

**S2** **Supplementary** **Materials and Methods**

**S2** Reagents

**S2** **Experimental Procedures**

**S2** Fluorescence-lifetime imaging microscopy

**S3 Supplementary Figures and Tables**

**S3** **Figure S1.** Image of photodegradation of internalized curcumin with antibiotics.

**S3** **Figure S2.** Spectral emission of curcumin without and with amoxicillin.

**S4** **Figure S3.** Decay time of curcumin without and with the presence of the antibiotic in the cell bacteria interior.

**S5 Supplementary Equations**

**S5** Calculation of intracellular concentration of curcumin at saturation

S6 **Supplementary Table**

S6 **Table S1.** Values in Log CFU/ml and standard deviation of combined treatments by oxidative stress (PDI/ANTB) and Simultaneous internalization (PDI+ANTB).

**Supplementary** **Materials and Methods**

**Reagents.** Amoxicillin, Erythromycin and Gentamicin sulfate was purchased from Chem-Impex®. Resazurin sodium salt (80%) used was from Oakwood®. NaCl, KCl, NaH_2_PO_4_ and KH_2_PO_4_ were acquired from Synth (Diadema, São Paulo, Brazil). The photosensitizer curcumin ((1*E*,6*E*)-1,7-bis-(4-hydroxy-3-methoxyphenyl)-1,6-heptadiene-3,5-dione) was supplied by PDT Pharma®. The culture media Mueller Hinton Broth No. 2 Control Cations was purchased from Himedia®.

**Experimental Procedures**

**Fluorescence-lifetime imaging microscopy (FLIM).** Fluorescence lifetime measurements were made during and after the curcumin (10 μM) internalization process, using the 2P laser. The images were collected in two different environments: aqueous solution deposited on the glass and in bacterial cell culture. The fluorescence was divided by a beam splitter in two detecting channels of a PicoQuant system: channel 1 detecting the fluorescence between 500 and 550 nm; and channel 2, detecting between 640 and 670 nm. The method used was the time correlated single photon counting (TCSPC) using avalanche detectors, which has a time response limited at about 100 ps. Two-exponential fit was used to adjust the fluorescence decay data. The choice of the fitting range was set by the software program (Time Trace Analysis by PicoQuant GmbH) by considering the decay part of the time dependent data according to optimal parameters. The optical setup was adjusted to the best signal-to-noise ratio and fixed when different samples were compared in both CLSM and FLIM modes.

**Supplementary Figures and Tables**

**
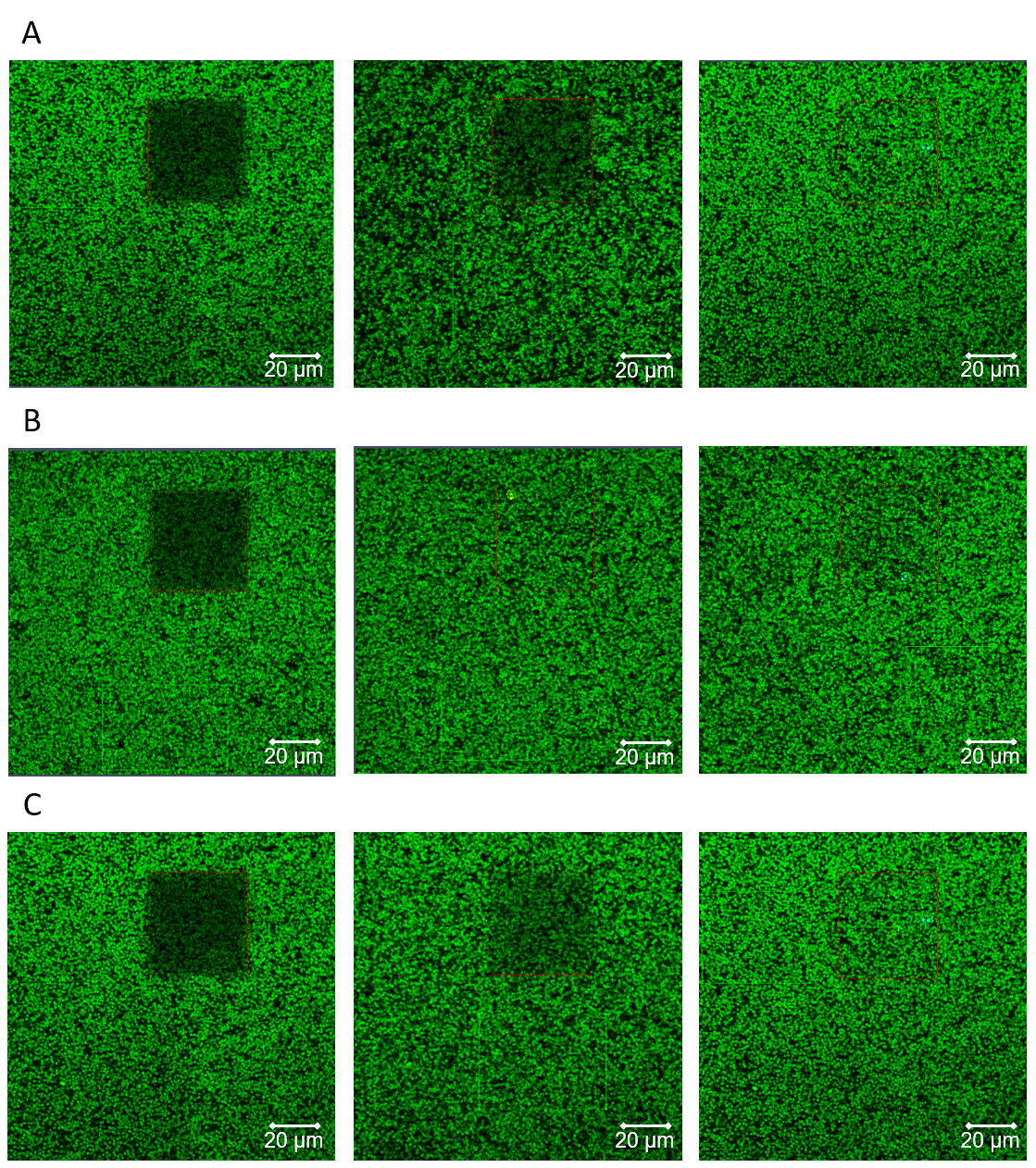
**

**Figure S1.** Confocal microscopy image of photodegradation and fluorescence recovery of curcumin internalized (10 μM) by *S. aureus* with the presence of the antibiotics amoxicillin, erythromycin and gentamicin Image o with A) amoxicillin, B) erythromycin and C) gentamicin. It was used 63x magnification and channel mode with timelapse


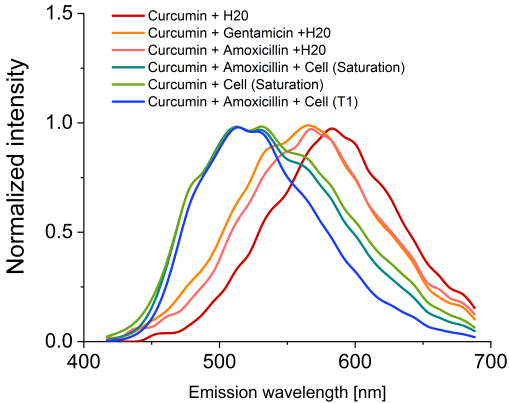


**Figure S2.** Spectral emission of curcumin without antibiotics (amoxicillin and gentamicin), in the absence and presence of *S. aureus*, at 10 min of uptake (T1), in the saturation of internalized curcumin (approximately 20 min of uptake).





**Figure S3.** Decay time of curcumin without and with the presence of the antibiotic (amoxicillin, erythromycin and gentamicin) in the cell interior, at 10 min of uptake (T1), in the saturation of internalized curcumin (approximately 20 min of uptake) and the Instrument Response Function (IRF).

**Supplementary Equations**

**Calculation of intracellular concentration of curcumin at saturation**

| Volume occupied by a molecule,  $V_{T}=\frac{4}{3}*\pi*R_{Trans}^{3}$ | Eq. S1 |
| --- | --- |
| $V_{T}=\frac{4}{3}*\pi*125\approx500nm^{3}$ | Eq S1.1 |
| Density of molecules,  $\frac{molécula}{V_{T}}=\frac{1}{500nm^{3}}=\frac{2*{10}^{-3}}{{10}^{-21}cm^{3}}=2*{10}^{19}\frac{moléculas}{ml}$ | Eq. S2 |
| Molar concentration,  $M=\frac{n}{V}=\frac{2*{10}^{19}}{6*{10}^{23}}=0,33\frac{mol}{ml}=33mM$ | Eq. S3 |

**Supplementary Table**

**Table S1.** Values in Log CFU/ml and standard deviation of combined treatments by oxidative stress (PDI/ANTB) and Simultaneous internalization (PDI+ANTB) with application of 10 μM of curcumin and 10 and 20 J/cm². The values correspond to the data shown in Figure 2 without the normalization of the survival fraction. The MIC value of each antibiotic is 0.25 µg/ml for AMO, 16 µg/ml for ERY and 0.5 µg/ml for GEN. * Values below the detection limit.

|  | Control | | PDI10 | | PDI20 | |  |  |  |  |  |  |
| --- | --- | --- | --- | --- | --- | --- | --- | --- | --- | --- | --- | --- |
|  | 9.55 | 0.6 | 7.50 | 1.5 | 7.48 | 1.1 |  |  |  |  |  |  |
|  |  |  |  |  |  |  |  |  |  |  |  |  |
|  | AMO | | ERY | | GEN | |  |  |  |  |  |  |
| 4MIC | 5.03 | 0.5 | 6.02 | 1.7 | 6.23 | 1.3 |  |  |  |  |  |  |
| 2MIC | 4.50 | 0.8 | 6.03 | 1.8 | 6.82 | 1.2 |  |  |  |  |  |  |
| MIC | 5.21 | 1.7 | 6.38 | 1.8 | 7.32 | 1.2 |  |  |  |  |  |  |
| 0.5MIC | 5.24 | 1.5 | 6.49 | 1.9 | 7.23 | 1.9 |  |  |  |  |  |  |
| 0.25MIC | 6.22 | 0.5 | 6.85 | 2.3 | 8.30 | 0.2 |  |  |  |  |  |  |
|  |  |  |  |  |  |  |  |  |  |  |  |  |
|  | 10PDI/AMO | | 20PDI/AMO | | 10PDI/ERY | | 20PDI/ERY | | 10PDI/GEN | | 20PDI/GEN | |
| 4MIC | 5.32 | 0.0 | 3.90 | 1.1 | 1.65 | 2.5 | 0.00* | 0 | 0.00* | 0 | 0.00* | 0 |
| 2MIC | 5.39 | 0.2 | 4.25 | 0.7 | 1.69 | 2.5 | 1.21 | 1.8 | 0.00* | 0 | 0.00* | 0 |
| MIC | 5.06 | 0.0 | 4.31 | 0.7 | 1.70 | 2.6 | 1.15 | 1.7 | 0.00* | 0 | 0.00* | 0 |
| 0.5MIC | 5.11 | 0.2 | 4.43 | 0.6 | 3.59 | 3.4 | 1.11 | 1.7 | 2.04 | 1.6 | 0.00* | 0 |
| 0.25MIC | 5.29 | 0.2 | 4.34 | 0.8 | 3.92 | 3.5 | 2.03 | 1.6 | 3.48 | 2.8 | 1.53 | 2.3 |
|  |  |  |  |  |  |  |  |  |  |  |  |  |
|  | 10PDI+AMO | | 20PDI+AMO | | 10PDI+ERY | | 20PDI+ERY | | 10PDI+GEN | | 10PDI+GEN | |
| 4MIC | 4.49 | 0.57 | 5.83 | 0.24 | 6.01 | 0.44 | 6.73 | 0.28 | 4.45 | 1.11 | 0.83 | 1.25 |
| 2MIC | 4.78 | 0.95 | 5.80 | 0.66 | 5.90 | 0.80 | 7.09 | 0.36 | 5.77 | 0.70 | 4.80 | 1.17 |
| MIC | 5.18 | 0.25 | 6.50 | 0.57 | 6.53 | 0.51 | 7.50 | 0.30 | 5.83 | 1.06 | 5.86 | 0.24 |
| 0.5MIC | 4.47 | 0.22 | 6.11 | 0.56 | 6.86 | 0.35 | 8.11 | 0.22 | 6.69 | 0.21 | 6.63 | 0.65 |
| 0.25MIC | 4.89 | 1.23 | 6.08 | 0.86 | 7.15 | 0.30 | 8.67 | 0.16 | 6.39 | 0.67 | 7.62 | 0.11 |
